# Nasal and pharyngeal carriage of methicillin-resistant *Staphylococcus sciuri* among hospitalised patients and healthcare workers in a Serbian university hospital

**DOI:** 10.1371/journal.pone.0185181

**Published:** 2017-09-19

**Authors:** Ivana Cirkovic, Jasmina Trajkovic, Tomasz Hauschild, Paal Skytt Andersen, Adebayo Shittu, Anders Rhod Larsen

**Affiliations:** 1 Institute of Microbiology and Immunology, Faculty of Medicine, University of Belgrade, Belgrade, Serbia; 2 Department of Microbiology, Institute of Biology, University of Bialystok, Bialystok, Poland; 3 Department Bacteria, Parasites and Fungi, Statens Serum Institut, Copenhagen, Denmark; 4 Department of Microbiology, Obafemi Awolowo University, Ile-Ife, Nigeria; Rockefeller University, UNITED STATES

## Abstract

There has been a paucity of data on methicillin-resistant *Staphylococcus sciuri* (MRSS) epidemiology in European healthcare settings. The aim of the study was to determine the prevalence of nasal and pharyngeal carriage and diversity of MRSS among inpatients and healthcare workers (HCWs) in the largest healthcare centre in Serbia, and to assess performance of different methods for MRSS screening. Nasal and pharyngeal swabs were obtained from 195 patients and 105 HCWs in different departments. Each swab was inoculated directly onto MRSA-ID, oxacillin-resistance screening agar and mannitol salt agar (MSA) with 2 mg/L of oxacillin. After inoculation, each swab was dipped in Mueller-Hinton broth with 6.5% NaCl and after overnight incubation, subcultured onto oxacillin-MSA. Characterisation of isolated MRSS strains was determined by antimicrobial susceptibility testing, PFGE, SCC*mec* typing and antimicrobial resistance genes detection. MRSS nasal and pharyngeal carriage rate was high (5%) in our hospital and department-variable. PFGE revealed a possible cross-transmission of MRSS between a patient and an HCW, and dissemination across hospital wards. All analysed isolates were multidrug resistant. Fusidic acid resistance was discovered in 93.7% of isolates, but *fusA* mutations in EF-G and *fusB/C* genes were not detected. SCC*mec* regions of MRSS contained elements of classic methicillin-resistant *S*. *aureus* type III. Broth enrichment prior to isolation on oxacillin-MSA was superior to direct cultivation on different media with a sensitivity/specificity of 100% and 88.5%, respectively. MRSS is a significant coloniser of patients and HCWs in the hospital. Further research is needed to investigate the clinical significance of the bacterium in our settings.

## Introduction

*Staphylococcus sciuri* belongs to the group of oxidase-positive, novobiocin-resistant coagulase-negative staphylococci (CoNS) [1]. This bacterium is widespread in nature and can be isolated from a variety of pets, wild and domestic animals, insects, environment (soil, sand, water, air samples, etc.), and foods [1–5]. It has also been recovered from the hospital environment [6] and although *S*. *sciuri* is only occasionally isolated from humans, it has been associated with a number of serious infections such as septicemia, endocarditis, peritonitis, pelvic inflammatory disease, urinary tract infections and wound infections [1,2,7,8]. An issue of particular interest is the frequent occurrence of genes conferring resistance to various antimicrobials in *S*. *sciuri* [3]. It has gained particular attention since it carries a *mecA* homologue (*mecA1*, *pbpD*) from which the *mecA* gene of methicillin-resistant staphylococci may have developed [3,9,10]. *mecA1* does not confer methicillin resistance in *S*. *sciuri*, but some strains may become resistant by acquisition of the *mecA* or *mecC* genes, identical to ones found in other staphylococci [3,7,11].

Methicillin-resistant *Staphylococcus aureus* (MRSA) has emerged as a major pathogen in the healthcare and community settings worldwide and where MRSA infections are often preceded by a period of carriage [12]. A number of studies on MRSA colonisation in hospitalised patients and healthcare workers (HCWs) have been performed all around world [12–14]. In a previous study, we found a high proportion of MRSA among patients and HCWs (10.3%) in a university hospital, whereby Serbia can be considered a country with a high prevalence of MRSA in the European perspective [12]. Still, the knowledge about carriage of other methicillin-resistant staphylococci in hospitals, including methicillin-resistant *S*. *sciuri* (MRSS), is scarce.

*S*. *sciuri* has long been the focus of our investigation [1–3,6,8], and during routine survey on the MRSA prevalence among hospitalised patients and HCWs in the hospital, we paid special attention to the presence of MRSS in the specimens. Here we present these results to provide baseline data on MRSS carriage among hospitalised patients and HCWs in the largest healthcare institution in Serbia and to evaluate the performance of different media for the detection of MRSS in screening samples.

## Materials and methods

### Study design, site and population

A cross-sectional study was conducted between November 2010 and January 2011 at the Clinical Centre of Serbia, which has over 3000 beds and represents the main referral healthcare centre in our country. Sample collections were performed in three departments: Emergency Department (ED), Surgical Department (SD) and Medical Department (MD).

Participation was voluntary and all participants signed informed consent form prior to their inclusion in the study. Descriptive information regarding participants' age, gender, diagnosis, department and period of hospitalisation was collected (S1 Table). The study protocol was approved by the Ethics Committee of the Faculty of Medicine, University of Belgrade, Belgrade, Serbia [no. 021/2010].

### Sampling

Specimens were taken from 195 hospitalised patients (mean age was 59.1+13.6; ages ranged from 19 to 85) and 105 HCWs (mean age was 46.3+11.1; ages ranged from 20 to 63). Samples for each person included two swabs, one taken from both anterior nares and one from the throat.

### Isolation and identification

After the collection, all samples were processed within 2 h. Each swab was inoculated directly onto MRSA-ID (bioMérieux, France), oxacillin resistance screening agar (ORSA; HiMedia, India) and mannitol salt agar (MSA; bioMérieux, France) supplemented with 2 mg/L of oxacillin. All inoculated media were incubated aerobically at 35°C and observed after 24 h and 48 h of incubation. The order of plating first alternated between the three media for every 100 samples. After inoculation, each swab was dipped in 3 mL of Mueller-Hinton broth (MHB; bioMérieux, France) supplemented with 6.5% NaCl and after incubation for 24 h at 35°C, subcultured onto MSA supplemented with 2 mg/L of oxacillin, which was thereafter incubated for up to 48 h at 35°C aerobically.

*S*. *sciuri* is a mannitol-positive organism [2,7], which shows alpha-glucosidase activity onMRSA-ID media. Therefore, mannitol-positive colonies on ORSA (blue colonies) and MSA (yellow colonies), as well as alpha-glucosidase positive colonies on MRSA-ID (green colonies) were subcultured onto trypticase soy agar (TSA; bioMérieux, France) and checked for oxidase activity. Identification of the strains was performed by previously described phenotypic methods [2] and confirmed by Vitek 2 system (bioMérieux). Methicillin resistance was confirmed by PCR for *mecA* gene [15]. *S*. *sciuri mecA* homologue was detected with *mecA* primers included in the PCR designed by Kondo et al. [16]. Strains *S*. *sciuri* subsp. *sciuri* CCM 3473, *S*. *sciuri* subsp. *rodentium* CCM 4657, *S*. *sciuri* subsp. *carnaticus* CCM 4835, *S*. *vitulinus* CCM 4511, *S*. *lentus* CCM 3472 and *S*. *aureus* ATCC 25923 were used as control.

### Antimicrobial susceptibility testing

Susceptibility to cefoxitin, chloramphenicol, ciprofloxacin, clindamycin, fusidic acid, erythromycin, gentamicin, kanamycin, linezolid, mupirocin, quinupristin-dalfopristin, penicillin, rifampin, tetracycline, tobramycin and trimethoprim/sulfamethoxazole (BioRad, USA) was tested by disk diffusion method and to vancomycin and teicoplanic by Etest (bioMérieux, France) in accordance with the European Committee on Antimicrobial Susceptibility Testing (EUCAST) recommendation (http://www.eucast.org). In order to identify MLSb phenotypes, a double-disk diffusion test (D test) was performed with erythromycin (15 μg) and clindamycin (2 μg), following the procedure recommended by EUCAST. Determination of minimum inhibitory concentration (MIC) of fusidic acid was performed by broth microdilution method in accordance with EUCAST recommendation. The molecular mechanism(s) of fusidic acid resistance in fusidic acid resistant isolates was investigated by testing isolates for the presence of the *fus*B and *fus*C genes using a multiplex PCR and by investigating isolates for the presence of *fus*A gene mutations by nucleotide sequencing [17].

All isolates were screened for aminoglycoside (*aacA-aphD*, *aphA*_3_, and *aadC*), macrolide-lincosamide-streptogramin (MLS) group of antibiotics (*erm*(A), *erm*(B), *erm*(C), *lnu*(A), *mph*(A), and *mph*(C)), tetracycline (*tet*(K), *tet*(M), *tet*(L) and *tet*(O)) and chloramphenicol (*cat*194, *cat*221 and *cat* 223) resistance genes [18–21].

### Molecular typing

PFGE was performed as described previously [6]. According to the criteria proposed by Tenover et al. [22], isolates whose PFGE pattern differed in more than six restriction fragments (bands) were genetically unrelated and were assigned to different pulsotypes (A-I). Isolates were considered to be closely related if their pulsotype differed in no more than three restriction bands and were assigned to pulsotype C1-C3.

Determination of SCC*mec* types was carried out by previously described PCR protocol for *mec* class and *ccr* type [16]. *S*. *aureus* strains HT20020290 (SCC*mec* type I), HT20020285 (SCC*mec* type II), HT20030826 (SCC*mec* type III), HT20040068 (SCC*mec* type IV), HT20060580 (SCC*mec* type V) and HT20020274 (SCC*mec* type VI), kindly provided by the Centre National de Référence des Staphylocoques, Lyon, France, were used as control. SCC*mec* typing was also performed on 37 MRSS strains previously isolated from humans, dogs, hospital environment and public transportation [1,2,6,8].

### Statistical analysis

Demographic characteristics (age and gender) of patients and HCWs and descriptive information regarding department, underlying diagnosis and duration of hospitalisation (Table 1, S1 Table) were described and compared by means of the χ2 test using SPSS 21+ statistical software.

**Table 1:** Distribution of methicillin-resistant *Staphylococcus sciuri* (MRSS) carriers and non-carriers among patients and healthcare workers (HCWs) stratified by department, gender, age group, patient underlying diagnosis and duration of hospitalisation at the Clinical Centre of Serbia, Serbia.

Stratifier	Group	Patients & HCWs (n = 300)		Patients (n = 195)		HCWs (n = 105)	
		Carrier	Non-carrier	Carrier	Non-carrier	Carrier	Non-carrier
Gender [n (%)]	Female	11 (6.2)	167 (93.7)	8 (10.3)	70 (89.7)	3 (3)	97 (97)
	Male	4 (3.3)	115 (96.7)	4 (3.4)	113 (96.6)	0 (0)	5 (100)
Department [n (%)]	ED	6 (6.3)	89 (93.7)	4 (3.4)	62 (93.9)	2(6.9)	27 (93.1)
	SD	8 (5.7)	132 (94.3)	7 (8.6)	74 (91.4)	1 (1.7)	58 (98.3)
	MD	1 (1.5)	64 (98.5)	1 (2.1)	47 (97.9)	0 (0)	17 (100)
Age group	< 65	14 (5.8)	229 (94.2)	11 (8)	127 (92)	3 (2.9)	102 (97.1)
	≥ 65	1(1.8)	56 (98.2)	1 (1.8)	56 (92.2)	/	/
Patient underlying diagnosis	Surgical	11 (8.3)	121 (91.7)	11 (8.3)	121 (91.7)	/	/
	Non-surgical	1 (1.6)	62 (98.4)	1 (1.6)	62 (98.4)		
Duration of hospitalisation [n (%)]	≤ 7 days	12 (7.9)	139 (92.1)	12 (7.9)	139 (92.1)	/	/
	> 7 days	0 (0)	44 (100)	0 (0)	44 (100)		

ED, Emergency Department; SD, Surgical Department; MD, Medical Department.

## Results

### Carriage rate

MRSS carrier prevalence in the two groups was 12/195 (6.1%) in patients and 3/105 (2.9%) in HCWs. Distribution of MRSS carriers and non-carriers stratified by population characteristics is shown in Table 1. Patients with surgical underlying diagnosis (p = 0.01), of female gender (p = 0.05) and hospitalised ≤ 7 days (p = 0.05) were more frequently colonised with MRSS.

The presence of MRSS in nasal and throat swab specimens obtained from patients and HCWs is presented in Table 2. The introduction of pharyngeal swabs in screening procedure increased MRSS carriage rate in patients by 16.7%, whereas in HCWs MRSS was recovered only from nasal samples.

**Table 2:** Presence of methicillin-resistant *Staphylococcus sciuri* in nasal and throat swab samples obtained from hospitalised patients and healthcare workers (HCWs).

Result for		Number (%) of individuals with result		
nasal specimen	throat specimen	Patients	HCWs	patients & HCWs
Positive	Positive	2 (1.0)*	-	2 (0.7)
Positive	Negative	8 (4.1)	3 (2.9)	11 (3.6)
Negative	Positive	2 (1.0)	-	2 (0.7)
Negative	Negative	183 (93.9)	102 (97.1)	285 (95.0)
Total positive		12 (6.1)	3 (2.9)	15 (5.0)

* In a nasal swab from one patient, two *S*. *sciuri* strains with different culture characteristics were isolated.

### Molecular typing and antimicrobial susceptibility

In total, 16 MRSS isolates were further analysed and the results of phenotypic and genotypic characterisation are presented in Table 3.

**Table 3:** Characteristics of methicillin-resistant *Staphylococcus sciuri* strains isolated from nasal and throat swab specimens obtained from hospitalised patients and healthcare workers (HCWs).

MRSS isolate	P/HCW	Department	Pulsotype	*mec* class	*ccr* type	Resistance profile	Resistance gene detected in isolate	MIC of fusidic acid (mg/L)
1	P	ED	A	A	3	GEN, KAN, TOB, FA, ERY, CLI, CIP, RIF	*aacA-aphD*, *aphA*_*3*,_ *erm*(C)	16
2	P	ED	B	A	3	GEN, KAN, TOB, FA, CLI(i)	*aacA-aphD*	4
3	P	SD	B	A	3	GEN, KAN, TOB, CLI(i)	*aacA-aphD*	1
4	P	SD	B	A	3	GEN, KAN, TOB, FA, CLI(i)	*aacA-aphD*	4
5	P	SD	B	A	3	GEN, KAN, TOB, FA, CLI(i)	*aacA-aphD*	4
6	P	ED	C1	A	NT	GEN, KAN, TOB, FA, CLI(i)	*aacA-aphD*, *aphA*_*3*,_ *lnu*(A)	16
7	HCW	ED	C2	A	3	GEN, KAN, TOB, FA, CLI(i)	*aacA-aphD*, *aphA*_*3*,_ *lnu*(A)	16
8	P	SD	C3	A	3	GEN, KAN, TOB, FA, CLI(i)	*aacA-aphD*, *aphA*_*3*,_ *erm*(C), *lnu*(A)	16
9	P	ED	D	A	NT	GEN, KAN, TOB, FA, CLI(i), CHL	*aacA-aphD*, *cat*221^1^	16
10	P	SD	E	A	3	GEN, KAN, TOB, FA, CLI(i)	*aacA-aphD*, *aadC*, *lnu*(A)	8
11	HCW	SD	E	A	3	GEN, KAN, TOB, FA, CLI(i)	*aacA-aphD*, *aadC*, *lnu*(A)	8
12	P	ED	F	A	3	GEN, KAN, TOB, FA, CLI(i), CHL	*aacA-aphD*, *aphA*_*3*,_ *cat*221^1^	8
13	P	SD	F	A	3	GEN, KAN, TOB, FA, CLI(i), CHL	*aacA-aphD*, *aphA*_*3*,_ *cat*221^1^	8
14	P	SD	G	A	NT	GEN, KAN, TOB, FA, CLI(i)	*aacA-aphD*, *aadC*	2
15	HCW	ED	H	A	3 + 5	GEN, KAN, TOB, CLI(i), TET, CIP. CHL, RIF(i), MUP	*aacA-aphD*, *aphA*_*3*,_ *cat*221, *tet*(M)	16
16	P	MD	I	A	NT	FA, CLI(i)	/	8

^1^, PCR product had a higher molecular weight than the positive control; P, patient; HCW, healthcare worker; NT, not typeable; ED, Emergency Department; SD, Surgical Department; MD, Medical Department, GEN, gentamicin; KAN, kanamycin; TOB, tobramycin; FA, fusidic acid; ERY, erythromycin; CLI, clindamycin; CIP, ciprofloxacin; RIF, rifampicin; TET, tetracycline; CHL, chloramphenicol; MUP, mupirocin; (i), intermediate resistant; MIC, minimum inhibitory concentration.

Based on PFGE typing, genetically unrelated MRSS could be divided into nine different pulsotypes (A-I). The largest group consisted of strains belonging to pulsotype B (25%) only found in patients from different departments (ED and SD).

SCC*mec* typing revealed that most of the MRSS strains (75%) from the study contained elements of classic SCC*mec* type III (*mec* class A and *ccr* type 3). Among 37 additional MRSS strains investigated from our collection, the combination of *mec* class A and *ccr* type 3 was detected in 17/37 (45.9%) strains, while only the presence of *mec* class A gene complex was identified in the remaining 20/37 (54.1%) strains.

All tested isolates were resistant to two or more antibiotics classes besides beta-lactam antibiotics, i.e. they were multidrug resistant. High levels of resistance were observed for aminoglycosides (93.8%) and fusidic acid (93.8%). Two (12.5%) and four (25%) isolates were resistant to ciprofloxacin and chloramphenicol, respectively. Only one isolate (6.3%) was resistant to rifampicin, tetracycline, mupirocin, erythromycin and clindamycin, while the remaining 15 (93.8%) strains exhibited intermediate resistance to clindamycin. All MRSS isolates were susceptible to trimethoprim/sulfamethoxazole, vancomycin, teicoplanin, linezolid and quinupristin-dalfopristin.

Of the 15 MRSS isolates that exhibited resistance to aminoglycosides, the *aacA-aphD*, *aphA*_*3*_ and *aadC* genes were detected in 100%, 46.7%, and 20% isolates, respectively. The *erm*(C) gene was responsible for the constitutive MLS_b_ resistance in 6.3% of isolates, whereas the *lnu*(A) gene was revealed in 31.3% of MRSS isolates. The *tet*(M) gene was identified in 6.3% of MRSS isolates that were also phenotypically resistant to tetracycline. The *cat*221 gene was discovered in a chloramphenicol-resistant isolate which was grouped in pulsotype D. However, in the remaining three isolates resistant to chloramphenicol (pulsotypes F and H), the PCR product with primers specific for *cat*221 were obtained, but the products had a higher molecular weight than the positive control.

MICs for fusidic acid showed low- to intermediate- resistance (2–16 mg/L), indicating presence of a *fusB* like resistance mechanisms. However, no amplification was obtained with the applied *fusB*/*fusC* PCR. Neither did sequencing of the EFG-G reveal mutations that correlated with the obtained MIC values. However, alterations in EFG-G defined two different clusters (A and B) and two single isolates with deviating amino acid substitutions (Table 4), whereas only substitution of K with N in position 342 (55-32-7 and 55-32-215) is a major alteration.

**Table 4:** Amino acid alterations in EFG-G of methicillin-resistant *Staphylococcus sciuri*.

Amino acid position	Cluster A	Cluster B	Isolate 55-32-7	Isolate 55-32-215
195	D	E	D	D
197	I	I	I	V
209	D	E	E	E
219	A	A	A	S
224	D	D	D	E
236	T	T	S	T
237	I	I	I	V
272	N	N	N	D
292	V	A	A	A
342	K	K	N	N
384	A	G	G	G

A, Alanine; D, Aspartate; E, Glutamate; G, Glycine; I, Isoleucine; K, Lysine; N, Asparagine; S, Serine; T, Threonine; V, Valine.

It was noticed that a pair of primers designed by Kondo et al. [16] to detect the *mecA* gene, also discover the presence of *S*. *sciuri mecA* homologue. The expected product of 286 bp was observed not only in isolated MRSS but also in methicillin-susceptible *S*. *sciuri* strains, including reference strains *S*. *sciuri* subsp. *sciuri* CCM 3473, *S*. *sciuri* subsp. *rodentium* CCM 4657 and *S*. *sciuri* subsp. *carnaticus* CCM 4835. Additionally, this 286 bp product was not obtained with *S*. *vitulinus* CCM 4511, *S*. *lentus* CCM 3472, or *S*. *aureus* ATCC 25923, which do not possess *mecA* homologue.

There was a good concordance between the observed antibiotic resistance profiles and the pulsotypes: pulsotype B and F (Table 3). Two *S*. *sciuri* isolates found in one swab taken from the anterior nares of a hospitalised patient were classified into pulsotypes B and G.

### Evaluation of methods for MRSS isolation

Table 5 summarises the results of the comparison of different methods and media used for MRSS isolation. Broth enrichment prior to isolation on MSA with oxacillin was superior to direct cultivation method on different media in detection of MRSS isolates with a sensitivity and specificity of 100% and 88.5% after 48h of incubation.

**Table 5:** Comparison of direct inoculation of MRSA-ID, ORSA, oxacillin-MSA and broth enrichment (BE) prior to inoculation on oxacillin-MSA in detection of methicillin-resistant *Staphylococcus sciuri* (MRSS) verified by identification by automated Vitek2 System and *mecA* PCR.

Medium	Number of MRSS strains detected (number after 48 h)	Sensitivity (%)		Specificity (%)	
		24 h	48 h	24 h	48 h
MRSA-ID	0 (2)	0	11.8	100	99.7
ORSA	0 (9)	0	52.9	99.1	97.0
O-MSA	1 (13)	5.9	76.5	98.9	95.7
BE + O-MSA	(17)	/	100	/	88.5

## Discussion

Isolation of *S*. *sciuri*, including methicillin-resistant strains, from nose of healthy and hospitalised individuals has previously been reported in low rates among healthy children (0.3%) [23], at hospital admission (0.4%) [24], or among persons in contact with horses (2.4%) [25]. To the best of our knowledge, this work represents the first comparison of MRSS strains isolated from patients and HCWs associated with the same ward/department during the same period. Carriage rate was surprisingly high in the Emergency (6.3%) and Surgical Departments (5.7%), but lower in the Medical Department (1.5%). Furthermore, the introduction of pharyngeal swabs in screening procedure increased MRSS carriage rate in patients by nearly 20%.

To determine the exact pattern of carriage in investigated individuals, non-carriers, intermittent carriers, or persistent carriers, longitudinal studies are required. However, persistent carriers are usually colonised by a higher load of a single strain over a long period, while intermittent carriers may carry different strains over time [26]. Semiquantitative analysis performed in our cross-sectional study, enabled us to conclude that most MRSS carriers were intermittent, because more than half of the strains were recovered from the samples after the broth enrichment step, indicating the presence of *S*. *sciuri* in very low numbers, while the remaining isolates produced only one to seven colonies during primary isolation.

As far as the origin of *S*. *sciuri* in humans is concerned, its transmission may occur via contact with animals. Another possible source for colonisation of humans with *S*. *sciuri* is food. In a previous study, a high rate of colonisation (10.5%) of hospital environment with *S*. *sciuri* was shown [6]. Among *S*. *sciuri* strains isolated from that hospital environment, 64.3% were resistant to methicillin. These results confirm the previous hypothesis of Couto et al. [9] that *S*. *sciuri* strains isolated from nasal passages and pharynx are acquired from the environment rather than from other sources. Yet, human to human transmission should also be considered.

PFGE analysis revealed nine pulsotypes within the population of MRSS strains. A high genetic diversity of *S*. *sciuri* strains isolated from hospital environment has also been reported in a previous study [6]. In the present investigation, isolation of MRSS with the same pulsotypes B and E from different individuals in different departments indicates possible dissemination of this bacterium between patients and hospital wards. In addition, MRSS strains with pulsotype E were isolated in the same department from a hospitalised patient and an HCW indicating a possible transmission between patient and HCW. A high resolution typing technique like whole genome sequencing would have been useful to substantiate this hypothesis.

Despite the fact that the clinical significance of *S*. *sciuri* may remain controversial (partially as the consequence of frequent non-identification of CoNS to the species level), the capacity of this bacterium to carry resistance determinants is well established [3,27]. This feature was confirmed in the present study, since resistance to various classes of antibiotics was detected in all tested MRSS strains. These strains probably acquired resistance to antibiotics in order to survive the high selective pressure in the hospital environment, where the antibiotic selective pressure is high. *S*. *sciuri* shows low level of natural resistance to lincosamides, i.e. licomycin and clindamycin [3], and therefore intermediate resistance to clindamycin was expected. However, it is interesting to note that 93.7% of strains were resistant to fusidic acid. This high proportion in *S*. *scuiri* isolates has also been confirmed in recent studies in animal species, pointing to a natural resistance of *S*. *scuiri* [3,28]. Fusidic acid resistance can either be caused by mutations in the EF-G-encoding *fusA* gene or by presence of the *fusB*, *fusC* and *fusD* genes which encode EF-G proteins protected from fusidic acid binding [3,27]. Therefore, we analysed fusidic acidresistant MRSS isolates for *fusA* mutations in EF-G, but could not find any. MICs of analysed isolates were in the low to intermediate range, pointing to a *fusB*-like mechanism, however *fusB/fusC* genes were not detected. Additionally, Schoenfelder *et al*. tested *S*. *sciuri* for *fusD* gene, which was also negative, indicating the presence of different mechanism mediating fusidic acid resistance that is still to be determined [3].

CoNS are believed to constitute a reservoir of SCC*mec* elements for *S*. *aureus*. Therefore, we analysed SCC*mec* regions of isolated MRSS strains. SCC*mec* typing revealed *mec* class A in all tested MRSS strains, but *ccr* type 3 was discovered in 12 (75%) strains. This *mec* class/*ccr* type combination corresponds to SCC*mec* type III [29]. In remaining four strains *ccr* type could not be determined. Presence of SCC*mec* type III or only *mec* class A in MRSS was also observed in other studies [30–32]. The obtained data suggest that all MRSS possess at least *mec* class complex A. Finally, we tested 37 previously described MRSS strains isolated from humans, dogs, hospital environment and public transportation [1,2,6,8]. SCC*mec* type III, i.e. combination of *mec* class A and *ccr* type 3, was detected in 45.9% of strains, while in the remaining strains only the presence of *mec* class A gene complex could be demonstrated. The high prevalence of non-typeable SCC*mec* cassettes in *S*. *sciuri* confirms the potential divergence of *ccr* and *mec* complexes as previously suggested [33] and indicates the presence of novel SCC*mec* elements [34].

*S*. *sciuri* is CoNS that can be easily misidentified as *S*. *aureus* because both species are mannitol-fermenting organisms that may grow as yellow colonies on blood agar and may give positive Slidex Staph Plus agglutination test and Staphaurex test [2,7]. Furthermore, MRSS can grow on chromogenic and selective media for MRSA strains giving the colonies of same colour, which is shown in our study. In accordance with this finding, the identity of isolated MRSA/MRSS strains on screening media has to be confirmed with biochemical tests (coagulase, oxidase and/or novobiocin susceptibility test) or in automated systems. Additionally, the importance of broth enrichment for accurate detection of MRSS in clinical sample is confirmed, as shown for MRSA [12].

## Conclusions

MRSS is a significant nasal and pharyngeal coloniser of patients and HCWs in this hospital. Molecular typing revealed the possibility of cross-transmission of MRSS between a patient and an HCW and dissemination through hospital. *S*. *scuiri* may serve as a reservoir and an important hub for exchange of the *mecA* gene and other resistance genes among staphylococci. In order to improve the detection of MRSS, broth enrichment prior to isolation on MSA with oxacillin represented the optimal available choice for isolating this bacterium.

## Supporting information

S1 Table Raw data. Distribution of MRSS carriers and non-carriers stratified by population characteristics and characteristics of MRSS strains isolated from carriers.
(DOCX)
