# Supplementary material for: Nasal and pharyngeal carriage of methicillin-resistant Staphylococcus sciuri among hospitalised patients and healthcare workers in a Serbian university hospital
Source: PLoS One. 2017 Sep 19;12(9):e0185181. doi: 10.1371/journal.pone.0185181 (PMC5605001; doi:10.1371/journal.pone.0185181)
Supplement: S1 Table — Distribution of MRSS carriers and non-carriers stratified by population characteristics and characteristics of MRSS strains isolated from carriers. (DOCX) [file pone.0185181.s001.docx]

Table S1. Raw data. Distribution of MRSS carriers and non-carriers stratified by population characteristics and characteristics of MRSS strains isolated from carriers

| Patient/HCW | Sex | Department | Age group (years) | Surgical/  Nonsurgical underlying diseases | Hospitalisation (days) | MRSS carriage status | Resistotype | Pulsotype | *mec* class | *ccr* type |
| --- | --- | --- | --- | --- | --- | --- | --- | --- | --- | --- |
| P 1 | F | ED | < 65 | S | < 7 | - |  |  |  |  |
| P 2 | F | ED | < 65 | S | < 7 | - |  |  |  |  |
| P 3 | F | ED | < 65 | S | < 7 | - |  |  |  |  |
| P 4 | F | ED | < 65 | S | < 7 | - |  |  |  |  |
| P 5 | F | ED | < 65 | S | < 7 | + | GEN, KAN, TOB, FA, ERY, CLI, CIP, RIF | A | A | 3 |
| P 6 | F | ED | < 65 | S | < 7 | - |  |  |  |  |
| P 7 | F | ED | < 65 | S | < 7 | - |  |  |  |  |
| P 8 | F | ED | < 65 | S | < 7 | + | GEN, KAN, TOB, FA, CLI(i), CHL | D | A | NT |
| P 9 | F | ED | < 65 | S | < 7 | - |  |  |  |  |
| P 10 | F | ED | < 65 | S | < 7 | - |  |  |  |  |
| P 11 | F | ED | < 65 | S | < 7 | - |  |  |  |  |
| P 12 | F | ED | > 65 | S | > 7 | - |  |  |  |  |
| P 13 | F | ED | > 65 | S | > 7 | - |  |  |  |  |
| P 14 | F | ED | > 65 | S | > 7 | - |  |  |  |  |
| P 15 | F | ED | > 65 | S | > 7 | - |  |  |  |  |
| P 16 | F | ED | > 65 | S | > 7 | - |  |  |  |  |
| P 17 | F | ED | < 65 | NS | < 7 | - |  |  |  |  |
| P 18 | F | ED | < 65 | NS | < 7 | - |  |  |  |  |
| P 19 | F | ED | < 65 | NS | < 7 | - |  |  |  |  |
| P 20 | F | ED | < 65 | NS | < 7 | - |  |  |  |  |
| P 21 | F | ED | < 65 | NS | < 7 | - |  |  |  |  |
| P 22 | F | ED | < 65 | NS | < 7 | - |  |  |  |  |
| P 23 | F | ED | > 65 | NS | > 7 | - |  |  |  |  |
| P 24 | F | ED | < 65 | S | < 7 | - |  |  |  |  |
| P 25 | F | ED | < 65 | S | < 7 | - |  |  |  |  |
| P 26 | F | ED | < 65 | S | < 7 | - |  |  |  |  |
| P 27 | F | ED | > 65 | S | < 7 | - |  |  |  |  |
| P 28 | F | SD | < 65 | S | < 7 | - |  |  |  |  |
| P 29 | F | SD | < 65 | S | < 7 | + | GEN, KAN, TOB, FA, CLI(i) | B | A | 3 |
| P 30 | F | SD | < 65 | S | < 7 | - |  |  |  |  |
| P 31 | F | SD | < 65 | S | < 7 | - |  |  |  |  |
| P 32 | F | SD | < 65 | S | < 7 | + | GEN, KAN, TOB, FA, CLI(i), CHL | F | A | 3 |
| P 33 | F | SD | < 65 | S | < 7 | - |  |  |  |  |
| P 34 | F | SD | < 65 | S | < 7 | - |  |  |  |  |
| P 35 | F | SD | < 65 | S | < 7 | - |  |  |  |  |
| P 36 | F | SD | < 65 | S | < 7 | - |  |  |  |  |
| P 37 | F | SD | < 65 | S | < 7 | + | GEN, KAN, TOB, FA, CLI(i) | B | A | 3 |
| P 38 | F | SD | < 65 | S | < 7 | - |  |  |  |  |
| P 39 | F | SD | < 65 | S | < 7 | + | GEN, KAN, TOB, FA, CLI(i) | E | A | 3 |
| P 40 | F | SD | < 65 | S | < 7 | - |  |  |  |  |
| P 41 | F | SD | < 65 | S | < 7 | + | GEN, KAN, TOB, FA, CLI(i) | C3 | A | 3 |
| P 42 | F | SD | > 65 | S | > 7 | - |  |  |  |  |
| P 43 | F | SD | > 65 | S | > 7 | - |  |  |  |  |
| P 44 | F | SD | > 65 | S | > 7 | - |  |  |  |  |
| P 45 | F | SD | > 65 | S | > 7 | - |  |  |  |  |
| P 46 | F | SD | > 65 | S | > 7 | - |  |  |  |  |
| P 47 | F | SD | > 65 | S | > 7 | - |  |  |  |  |
| P 48 | F | SD | > 65 | S | > 7 | - |  |  |  |  |
| P 49 | F | SD | < 65 | NS | < 7 | - |  |  |  |  |
| P 50 | F | SD | < 65 | NS | < 7 | - |  |  |  |  |
| P 51 | F | SD | < 65 | NS | < 7 | - |  |  |  |  |
| P 52 | F | SD | < 65 | NS | < 7 | - |  |  |  |  |
| P 53 | F | SD | < 65 | NS | < 7 | - |  |  |  |  |
| P 54 | F | SD | < 65 | NS | < 7 | - |  |  |  |  |
| P 55 | F | SD | < 65 | NS | < 7 | - |  |  |  |  |
| P 56 | F | SD | < 65 | NS | < 7 | - |  |  |  |  |
| P 57 | F | SD | < 65 | NS | < 7 | - |  |  |  |  |
| P 58 | F | SD | > 65 | NS | > 7 | - |  |  |  |  |
| P 59 | F | SD | > 65 | S | < 7 | - |  |  |  |  |
| P 60 | F | SD | > 65 | S | < 7 | - |  |  |  |  |
| P 61 | F | SD | < 65 | NS | < 7 | - |  |  |  |  |
| P 62 | F | MD | > 65 | S | < 7 | - |  |  |  |  |
| P 63 | F | MD | > 65 | S | < 7 | - |  |  |  |  |
| P 64 | F | MD | > 65 | S | < 7 | - |  |  |  |  |
| P 65 | F | MD | > 65 | S | < 7 | - |  |  |  |  |
| P 66 | F | MD | > 65 | S | < 7 | - |  |  |  |  |
| P 67 | F | MD | > 65 | S | < 7 | - |  |  |  |  |
| P 68 | F | MD | < 65 | S | > 7 | - |  |  |  |  |
| P 69 | F | MD | < 65 | S | > 7 | - |  |  |  |  |
| P 70 | F | MD | < 65 | S | > 7 | - |  |  |  |  |
| P 71 | F | MD | > 65 | NS | < 7 | - |  |  |  |  |
| P 72 | F | MD | > 65 | NS | < 7 | - |  |  |  |  |
| P 73 | F | MD | > 65 | NS | < 7 | - |  |  |  |  |
| P 74 | F | MD | > 65 | NS | < 7 | - |  |  |  |  |
| P 75 | F | MD | > 65 | NS | < 7 | - |  |  |  |  |
| P 76 | F | MD | > 65 | NS | < 7 | + | FA, CLI(i) | I | A | NT |
| P 77 | F | MD | < 65 | NS | > 7 | - |  |  |  |  |
| P 78 | F | MD | > 65 | NS | < 7 | - |  |  |  |  |
| P 79 | F | MD | > 65 | NS | > 7 | - |  |  |  |  |
| P 80 | M | ED | < 65 | S | < 7 | + | GEN, KAN, TOB, FA, CLI(i) | B | A | 3 |
| P 80 | M | ED | < 65 | S | < 7 | + | GEN, KAN, TOB, FA, CLI(i), CHL | F | A | 3 |
| P 81 | M | ED | < 65 | S | < 7 | - |  |  |  |  |
| P 82 | M | ED | < 65 | S | < 7 | - |  |  |  |  |
| P 83 | M | ED | < 65 | S | < 7 | - |  |  |  |  |
| P 84 | M | ED | < 65 | S | < 7 | - |  |  |  |  |
| P 85 | M | ED | < 65 | S | < 7 | - |  |  |  |  |
| P 86 | M | ED | < 65 | S | < 7 | - |  |  |  |  |
| P 87 | M | ED | < 65 | S | < 7 | - |  |  |  |  |
| P 88 | M | ED | < 65 | S | < 7 | + | GEN, KAN, TOB, FA, CLI(i) | C1 | A | NT |
| P 89 | M | ED | < 65 | S | < 7 | - |  |  |  |  |
| P 90 | M | ED | < 65 | S | < 7 | - |  |  |  |  |
| P 91 | M | ED | < 65 | S | < 7 | - |  |  |  |  |
| P 92 | M | ED | < 65 | S | < 7 | - |  |  |  |  |
| P 93 | M | ED | < 65 | S | < 7 | - |  |  |  |  |
| P 94 | M | ED | > 65 | S | > 7 | - |  |  |  |  |
| P 95 | M | ED | > 65 | S | > 7 | - |  |  |  |  |
| P 96 | M | ED | > 65 | S | > 7 | - |  |  |  |  |
| P 97 | M | ED | > 65 | S | > 7 | - |  |  |  |  |
| P 98 | M | ED | < 65 | NS | < 7 | - |  |  |  |  |
| P 99 | M | ED | < 65 | NS | < 7 | - |  |  |  |  |
| P 100 | M | ED | < 65 | NS | < 7 | - |  |  |  |  |
| P 101 | M | ED | < 65 | NS | < 7 | - |  |  |  |  |
| P 102 | M | ED | < 65 | NS | < 7 | - |  |  |  |  |
| P 103 | M | ED | < 65 | NS | < 7 | - |  |  |  |  |
| P 104 | M | ED | < 65 | NS | < 7 | - |  |  |  |  |
| P 105 | M | ED | < 65 | NS | < 7 | - |  |  |  |  |
| P 106 | M | ED | > 65 | NS | > 7 | - |  |  |  |  |
| P 107 | M | ED | > 65 | NS | > 7 | - |  |  |  |  |
| P 108 | M | ED | < 65 | S | < 7 | - |  |  |  |  |
| P 109 | M | ED | < 65 | S | < 7 | - |  |  |  |  |
| P 110 | M | ED | < 65 | S | < 7 | - |  |  |  |  |
| P 111 | M | ED | < 65 | S | < 7 | - |  |  |  |  |
| P 112 | M | ED | < 65 | S | < 7 | - |  |  |  |  |
| P 113 | M | ED | < 65 | S | < 7 | - |  |  |  |  |
| P 114 | M | ED | < 65 | S | < 7 | - |  |  |  |  |
| P 115 | M | ED | < 65 | S | < 7 | - |  |  |  |  |
| P 116 | M | ED | < 65 | NS | < 7 | - |  |  |  |  |
| P 117 | M | ED | < 65 | NS | < 7 | - |  |  |  |  |
| P 118 | M | ED | < 65 | NS | < 7 | - |  |  |  |  |
| P 119 | M | SD | < 65 | S | < 7 | - |  |  |  |  |
| P 120 | M | SD | < 65 | S | < 7 | - |  |  |  |  |
| P 121 | M | SD | < 65 | S | < 7 | - |  |  |  |  |
| P 122 | M | SD | < 65 | S | < 7 | - |  |  |  |  |
| P 123 | M | SD | < 65 | S | < 7 | - |  |  |  |  |
| P 124 | M | SD | < 65 | S | < 7 | - |  |  |  |  |
| P 125 | M | SD | < 65 | S | < 7 | - |  |  |  |  |
| P 126 | M | SD | < 65 | S | < 7 | - |  |  |  |  |
| P 127 | M | SD | < 65 | S | < 7 | + | GEN, KAN, TOB, CLI(i) | B | A | 3 |
| P 128 | M | SD | < 65 | S | < 7 | - |  |  |  |  |
| P 129 | M | SD | < 65 | S | < 7 | - |  |  |  |  |
| P 130 | M | SD | < 65 | S | < 7 | - |  |  |  |  |
| P 131 | M | SD | < 65 | S | < 7 | - |  |  |  |  |
| P 132 | M | SD | < 65 | S | < 7 | - |  |  |  |  |
| P 133 | M | SD | < 65 | S | < 7 | - |  |  |  |  |
| P 134 | M | SD | < 65 | S | < 7 | - |  |  |  |  |
| P 135 | M | SD | < 65 | S | < 7 | - |  |  |  |  |
| P 136 | M | SD | < 65 | S | < 7 | - |  |  |  |  |
| P 137 | M | SD | < 65 | S | < 7 | - |  |  |  |  |
| P 138 | M | SD | < 65 | S | < 7 | - |  |  |  |  |
| P 139 | M | SD | < 65 | S | < 7 | - |  |  |  |  |
| P 140 | M | SD | < 65 | S | < 7 | - |  |  |  |  |
| P 141 | M | SD | < 65 | S | < 7 | - |  |  |  |  |
| P 142 | M | SD | < 65 | S | < 7 | - |  |  |  |  |
| P 143 | M | SD | < 65 | S | < 7 | + | GEN, KAN, TOB, FA, CLI(i) | G | A | NT |
| P 144 | M | SD | > 65 | S | > 7 | - |  |  |  |  |
| P 145 | M | SD | > 65 | S | > 7 | - |  |  |  |  |
| P 146 | M | SD | > 65 | S | > 7 | - |  |  |  |  |
| P 147 | M | SD | > 65 | S | > 7 | - |  |  |  |  |
| P 148 | M | SD | > 65 | S | > 7 | - |  |  |  |  |
| P 149 | M | SD | > 65 | S | > 7 | - |  |  |  |  |
| P 150 | M | SD | > 65 | S | > 7 | - |  |  |  |  |
| P 151 | M | SD | < 65 | NS | < 7 | - |  |  |  |  |
| P 152 | M | SD | < 65 | NS | < 7 | - |  |  |  |  |
| P 153 | M | SD | < 65 | NS | < 7 | - |  |  |  |  |
| P 154 | M | SD | < 65 | NS | < 7 | - |  |  |  |  |
| P 155 | M | SD | < 65 | NS | < 7 | - |  |  |  |  |
| P 156 | M | SD | < 65 | NS | < 7 | - |  |  |  |  |
| P 157 | M | SD | < 65 | NS | < 7 | - |  |  |  |  |
| P 158 | M | SD | < 65 | NS | < 7 | - |  |  |  |  |
| P 159 | M | SD | < 65 | NS | < 7 | - |  |  |  |  |
| P 160 | M | SD | < 65 | NS | < 7 | - |  |  |  |  |
| P 161 | M | SD | > 65 | NS | > 7 | - |  |  |  |  |
| P 162 | M | SD | > 65 | NS | > 7 | - |  |  |  |  |
| P 163 | M | SD | > 65 | NS | > 7 | - |  |  |  |  |
| P 164 | M | SD | < 65 | S | < 7 | - |  |  |  |  |
| P 165 | M | SD | < 65 | S | < 7 | - |  |  |  |  |
| P 166 | M | MD | > 65 | NS | > 7 | - |  |  |  |  |
| P 167 | M | MD | < 65 | S | < 7 | - |  |  |  |  |
| P 168 | M | MD | < 65 | S | < 7 | - |  |  |  |  |
| P 169 | M | MD | < 65 | S | < 7 | - |  |  |  |  |
| P 170 | M | MD | < 65 | S | < 7 | - |  |  |  |  |
| P 171 | M | MD | < 65 | S | < 7 | - |  |  |  |  |
| P 172 | M | MD | < 65 | S | < 7 | - |  |  |  |  |
| P 173 | M | MD | < 65 | S | < 7 | - |  |  |  |  |
| P 174 | M | MD | < 65 | S | < 7 | - |  |  |  |  |
| P 175 | M | MD | < 65 | S | < 7 | - |  |  |  |  |
| P 176 | M | MD | < 65 | S | < 7 | - |  |  |  |  |
| P 177 | M | MD | < 65 | S | < 7 | - |  |  |  |  |
| P 178 | M | MD | < 65 | S | < 7 | - |  |  |  |  |
| P 179 | M | MD | < 65 | S | < 7 | - |  |  |  |  |
| P 180 | M | MD | < 65 | S | < 7 | - |  |  |  |  |
| P 181 | M | MD | < 65 | S | < 7 | - |  |  |  |  |
| P 182 | M | MD | < 65 | S | < 7 | - |  |  |  |  |
| P 183 | M | MD | > 65 | S | > 7 | - |  |  |  |  |
| P 184 | M | MD | > 65 | S | > 7 | - |  |  |  |  |
| P 185 | M | MD | > 65 | S | > 7 | - |  |  |  |  |
| P 186 | M | MD | > 65 | S | > 7 | - |  |  |  |  |
| P 187 | M | MD | > 65 | NS | < 7 | - |  |  |  |  |
| P 188 | M | MD | > 65 | NS | < 7 | - |  |  |  |  |
| P 189 | M | MD | > 65 | NS | < 7 | - |  |  |  |  |
| P 190 | M | MD | > 65 | NS | < 7 | - |  |  |  |  |
| P 191 | M | MD | > 65 | NS | < 7 | - |  |  |  |  |
| P 192 | M | MD | > 65 | NS | < 7 | - |  |  |  |  |
| P 193 | M | MD | < 65 | NS | > 7 | - |  |  |  |  |
| P 194 | M | MD | < 65 | NS | > 7 | - |  |  |  |  |
| P 195 | M | MD | < 65 | NS | > 7 | - |  |  |  |  |
| HCW1 | M | ED | < 65 | / | / | - |  |  |  |  |
| HCW2 | M | ED | < 65 | / | / | - |  |  |  |  |
| HCW3 | M | ED | < 65 | / | / | - |  |  |  |  |
| HCW4 | M | ED | < 65 | / | / | - |  |  |  |  |
| HCW5 | M | ED | < 65 | / | / | - |  |  |  |  |
| HCW6 | F | ED | < 65 | / | / | - |  |  |  |  |
| HCW7 | F | ED | < 65 | / | / | - |  |  |  |  |
| HCW8 | F | ED | < 65 | / | / | - |  |  |  |  |
| HCW9 | F | ED | < 65 | / | / | - |  |  |  |  |
| HCW10 | F | ED | < 65 | / | / | - |  |  |  |  |
| HCW11 | F | ED | < 65 | / | / | - |  |  |  |  |
| HCW12 | F | ED | < 65 | / | / | - |  |  |  |  |
| HCW13 | F | ED | < 65 | / | / | - |  |  |  |  |
| HCW14 | F | ED | < 65 | / | / | - |  |  |  |  |
| HCW15 | F | ED | < 65 | / | / | - |  |  |  |  |
| HCW16 | F | ED | < 65 | / | / | - |  |  |  |  |
| HCW17 | F | ED | < 65 | / | / | - |  |  |  |  |
| HCW18 | F | ED | < 65 | / | / | + | GEN, KAN, TOB, FA, CLI(i) | C2 | A | 3 |
| HCW19 | F | ED | < 65 | / | / | - |  |  |  |  |
| HCW20 | F | ED | < 65 | / | / | - |  |  |  |  |
| HCW21 | F | ED | < 65 | / | / | - |  |  |  |  |
| HCW22 | F | ED | < 65 | / | / | - |  |  |  |  |
| HCW23 | F | ED | < 65 | / | / | - |  |  |  |  |
| HCW24 | F | ED | < 65 | / | / | - |  |  |  |  |
| HCW25 | F | ED | < 65 | / | / | - |  |  |  |  |
| HCW26 | F | ED | < 65 | / | / | - |  |  |  |  |
| HCW27 | F | ED | < 65 | / | / | + | GEN, KAN, TOB, CLI(i), TET, CIP. CHL, RIF(i), MUP | H | A | 3 + 5 |
| HCW28 | F | ED | < 65 | - | - | - |  |  |  |  |
| HCW29 | F | ED | < 65 | - | - | - |  |  |  |  |
| HCW30 | F | SD | < 65 | / | / | - |  |  |  |  |
| HCW31 | F | SD | < 65 | / | / | - |  |  |  |  |
| HCW32 | F | SD | < 65 | / | / | - |  |  |  |  |
| HCW33 | F | SD | < 65 | / | / | - |  |  |  |  |
| HCW34 | F | SD | < 65 | / | / | - |  |  |  |  |
| HCW35 | F | SD | < 65 | / | / | - |  |  |  |  |
| HCW36 | F | SD | < 65 | / | / | - |  |  |  |  |
| HCW37 | F | SD | < 65 | / | / | - |  |  |  |  |
| HCW38 | F | SD | < 65 | / | / | - |  |  |  |  |
| HCW39 | F | SD | < 65 | / | / | - |  |  |  |  |
| HCW40 | F | SD | < 65 | / | / | - |  |  |  |  |
| HCW41 | F | SD | < 65 | / | / | + | GEN, KAN, TOB, FA, CLI(i) | E | A | 3 |
| HCW42 | F | SD | < 65 | / | / | - |  |  |  |  |
| HCW43 | F | SD | < 65 | / | / | - |  |  |  |  |
| HCW44 | F | SD | < 65 | / | / | - |  |  |  |  |
| HCW45 | F | SD | < 65 | / | / | - |  |  |  |  |
| HCW46 | F | SD | < 65 | / | / | - |  |  |  |  |
| HCW47 | F | SD | < 65 | / | / | - |  |  |  |  |
| HCW48 | F | SD | < 65 | / | / | - |  |  |  |  |
| HCW49 | F | SD | < 65 | / | / | - |  |  |  |  |
| HCW50 | F | SD | < 65 | / | / | - |  |  |  |  |
| HCW51 | F | SD | < 65 | / | / | - |  |  |  |  |
| HCW52 | F | SD | < 65 | / | / | - |  |  |  |  |
| HCW53 | F | SD | < 65 | / | / | - |  |  |  |  |
| HCW54 | F | SD | < 65 | / | / | - |  |  |  |  |
| HCW55 | F | SD | < 65 | / | / | - |  |  |  |  |
| HCW56 | F | SD | < 65 | / | / | - |  |  |  |  |
| HCW57 | F | SD | < 65 | / | / | - |  |  |  |  |
| HCW58 | F | SD | < 65 | / | / | - |  |  |  |  |
| HCW59 | F | SD | < 65 | / | / | - |  |  |  |  |
| HCW60 | F | SD | < 65 | / | / | - |  |  |  |  |
| HCW61 | F | SD | < 65 | / | / | - |  |  |  |  |
| HCW62 | F | SD | < 65 | / | / | - |  |  |  |  |
| HCW63 | F | SD | < 65 | / | / | - |  |  |  |  |
| HCW64 | F | SD | < 65 | / | / | - |  |  |  |  |
| HCW65 | F | SD | < 65 | / | / | - |  |  |  |  |
| HCW66 | F | SD | < 65 | / | / | - |  |  |  |  |
| HCW67 | F | SD | < 65 | / | / | - |  |  |  |  |
| HCW68 | F | SD | < 65 | / | / | - |  |  |  |  |
| HCW69 | F | SD | < 65 | / | / | - |  |  |  |  |
| HCW70 | F | SD | < 65 | / | / | - |  |  |  |  |
| HCW71 | F | SD | < 65 | / | / | - |  |  |  |  |
| HCW72 | F | SD | < 65 | / | / | - |  |  |  |  |
| HCW73 | F | SD | < 65 | / | / | - |  |  |  |  |
| HCW74 | F | SD | < 65 | / | / | - |  |  |  |  |
| HCW75 | F | SD | < 65 | / | / | - |  |  |  |  |
| HCW76 | F | SD | < 65 | / | / | - |  |  |  |  |
| HCW77 | F | SD | < 65 | / | / | - |  |  |  |  |
| HCW78 | F | SD | < 65 | / | / | - |  |  |  |  |
| HCW79 | F | SD | < 65 | / | / | - |  |  |  |  |
| HCW80 | F | SD | < 65 | / | / | - |  |  |  |  |
| HCW81 | F | SD | < 65 | / | / | - |  |  |  |  |
| HCW82 | F | SD | < 65 | / | / | - |  |  |  |  |
| HCW83 | F | SD | < 65 | / | / | - |  |  |  |  |
| HCW84 | F | SD | < 65 | - | - | - |  |  |  |  |
| HCW85 | F | SD | < 65 | - | - | - |  |  |  |  |
| HCW86 | F | SD | < 65 | - | - | - |  |  |  |  |
| HCW87 | F | SD | < 65 | - | - | - |  |  |  |  |
| HCW88 | F | SD | < 65 | - | - | - |  |  |  |  |
| HCW89 | F | SD | < 65 | - | - | - |  |  |  |  |
| HCW90 | F | MD | < 65 | / | / | - |  |  |  |  |
| HCW91 | F | MD | < 65 | / | / | - |  |  |  |  |
| HCW92 | F | MD | < 65 | / | / | - |  |  |  |  |
| HCW93 | F | MD | < 65 | / | / | - |  |  |  |  |
| HCW94 | F | MD | < 65 | / | / | - |  |  |  |  |
| HCW95 | F | MD | < 65 | / | / | - |  |  |  |  |
| HCW96 | F | MD | < 65 | / | / | - |  |  |  |  |
| HCW97 | F | MD | < 65 | / | / | - |  |  |  |  |
| HCW98 | F | MD | < 65 | / | / | - |  |  |  |  |
| HCW99 | F | MD | < 65 | / | / | - |  |  |  |  |
| HCW100 | F | MD | < 65 | / | / | - |  |  |  |  |
| HCW101 | F | MD | < 65 | / | / | - |  |  |  |  |
| HCW102 | F | MD | < 65 | / | / | - |  |  |  |  |
| HCW103 | F | MD | < 65 | / | / | - |  |  |  |  |
| HCW104 | F | MD | < 65 | / | / | - |  |  |  |  |
| HCW105 | F | MD | < 65 | / | / | - |  |  |  |  |

P, patient; HCW, healthcare worker; F, female; M, male; ED, Emergency Department; SD, Surgical Department; MD, Medical Department; S, surgical; NS, nonsurgical; NT, not typeable; GEN, gentamicin; KAN, kanamycin; TOB, tobramycin; FA, fusidic acid; ERY, erythromycin; CLI, clindamycin; CIP, ciprofloxacin; RIF, rifampicin; TET, tetracycline; CHL, chloramphenicol; MUP, mupirocin; (i), intermediate resistant.
